# Someone like me: Size-assortative pairing and mating in an Amazonian fish, sailfin tetra *Crenuchus spilurus*

**DOI:** 10.1371/journal.pone.0222880

**Published:** 2019-09-27

**Authors:** Elio de Almeida Borghezan, Kalebe da Silva Pinto, Jansen Zuanon, Tiago Henrique da Silva Pires

**Affiliations:** 1 Laboratório de Ecologia Comportamental e Evolução, Instituto Nacional de Pesquisas da Amazônia, Av. André Araújo, Manaus, AM, Brazil; 2 Wildlife Research Center of Kyoto University, Sakyo-ku, Kyoto, Japan; Institute of Marine Research, NORWAY

## Abstract

In the absence of constraints, preference for larger mates is expected to evolve, as larger individuals are typical of higher potential fitness. Large females are often more fecund and carry larger eggs (which result in higher number and better quality of offspring), whereas large males usually have more conspicuous ornaments and are better at defending resources. However, intrasexual competition can constrain the access to larger partners, especially when opportunities for mate takeover abound. Here we investigate the relationship between individual’s size and mate choice in relation to one’s own size and their respective mate’s size using the sailfin tetra, a sexually dimorphic Amazonian fish species. We show that ornaments of larger males are exponentially more conspicuous, and larger females are more fecund and carry larger eggs. Contrary to expectation, neither males nor females associated for longer with the larger of two offered potential mates. Instead, individuals of both genders chose opposite-sex individuals of similar sizes to themselves. Additionally, similar-sized pairs were more likely to spawn than couples with higher size asymmetries. Grounded on field observations, we propose that prudent choice should be particularly important in this system, since courtship is long (often taking several days), which offers opportunities for mate takeover. Intrasexual competition, however, cannot readily explain female choice for similar-sized males. We thus suggest that such preference might be best explained by avoidance of filial cannibalism.

## Introduction

The correlation between traits of males and females in mated pairs is commonly observed both in nature and in controlled experiments [1–5]. Known as assortative mating, this association can be based on a great variety of phenotypic characteristics [3]. Paired individuals can be similar in color [4,6], age [7,8], quality [9], or size [2,5]. Similarity in sizes of paired males and females during mating is commonly reported and occurs in organisms ranging from earthworms [10], insects [2,11], spiders [12], crustaceans [13], fishes [1], birds [14], and mammals [15].

Size-assortative mating may result from several processes, some not related to the active choice of partners [16,17]. Also, size assortative mating may be observed when individuals do not prefer partners that are similar to themselves [1,5,16–18]. However, several cases have been reported in which individuals exert active preference for mating partners of similar sizes [2,12,13].

Due to their higher parental investments, females should choose mates from multiple potential males [19,20]. Favoring larger males may yield multiple benefits to the choosing female, as larger males typically have higher competitive ability (or, resource holding potential), often observed in their higher likelihood of winning dyadic encounters [17,21,22], in being better at maintaining larger and better territories [23,24], in successfully defending the offspring against conspecifics [25–27], or in being more capable of providing offspring with high-quality parental care, which include spending more time guarding the offspring, higher frequency of fanning and nest cleaning behavior [27,28]. Female choice may also be purely aesthetic, as originally suggested by Charles Darwin [29,30]. Under purely aesthetic preference, larger males might be preferred, since they typically bear exponentially more conspicuous ornaments. Conversely, larger ornaments might exploit female choice for male size [31] and males with larger and more conspicuous ornaments may be favored by females, even at potential viability cost for the bearer [32]. Additionally, because conspicuousness of ornaments often increases as males grow, so does the quality of signals emitted to females [33].

Males are expected to be selective for females when finding mates is not energetically demanding and when females vary highly in quality [34,35]. Male choice is also expected to occur when males contribute with a high investment to the mating partner and/or the offspring [19,36], as they might be unavailable for mating for a long period, penalizing their potential reproductive rate [37], a pattern common in species with exclusive paternal care [19]. Since fecundity and egg size are often related to female body size [38,39], males often benefit from preferring larger females as mates [13,40–43]. Therefore, due to their high energetic expenditures in mating investment and parental care, both females and males are expected to choose larger mates in sexually dimorphic species with exclusive paternal care.

However, when individuals of one or both sexes prefer to mate with larger (and higher quality) mates, preference for larger mate size might be constrained by intrasexual competition [2,5,13,44,45]. Under the scenario of strong competition for larger mates, smaller individuals might not be able to maintain high quality mates due to mate takeover [46–48], which may result in smaller or poor-quality individuals mating with smaller or lower quality mates [9,48]. When mate takeover is common, sexual selection should favor individuals that avoid investing time and effort in high quality mates they cannot maintain, resulting in preference for similar-sized partners [13,47,49,50]. This phenomenon has been termed prudent choice [51–53].

Because prudent mate choice occurs via takeover, it can be particularly important in species whose courtship or mate guarding until fertilization is long lasting [13,48,50]. Pairing of couples can last for several days, or even weeks, in numerous organisms [54–57]. Under this scenario, opportunities are increased for larger males to potentially takeover larger females from smaller males.

**Fig 1:**
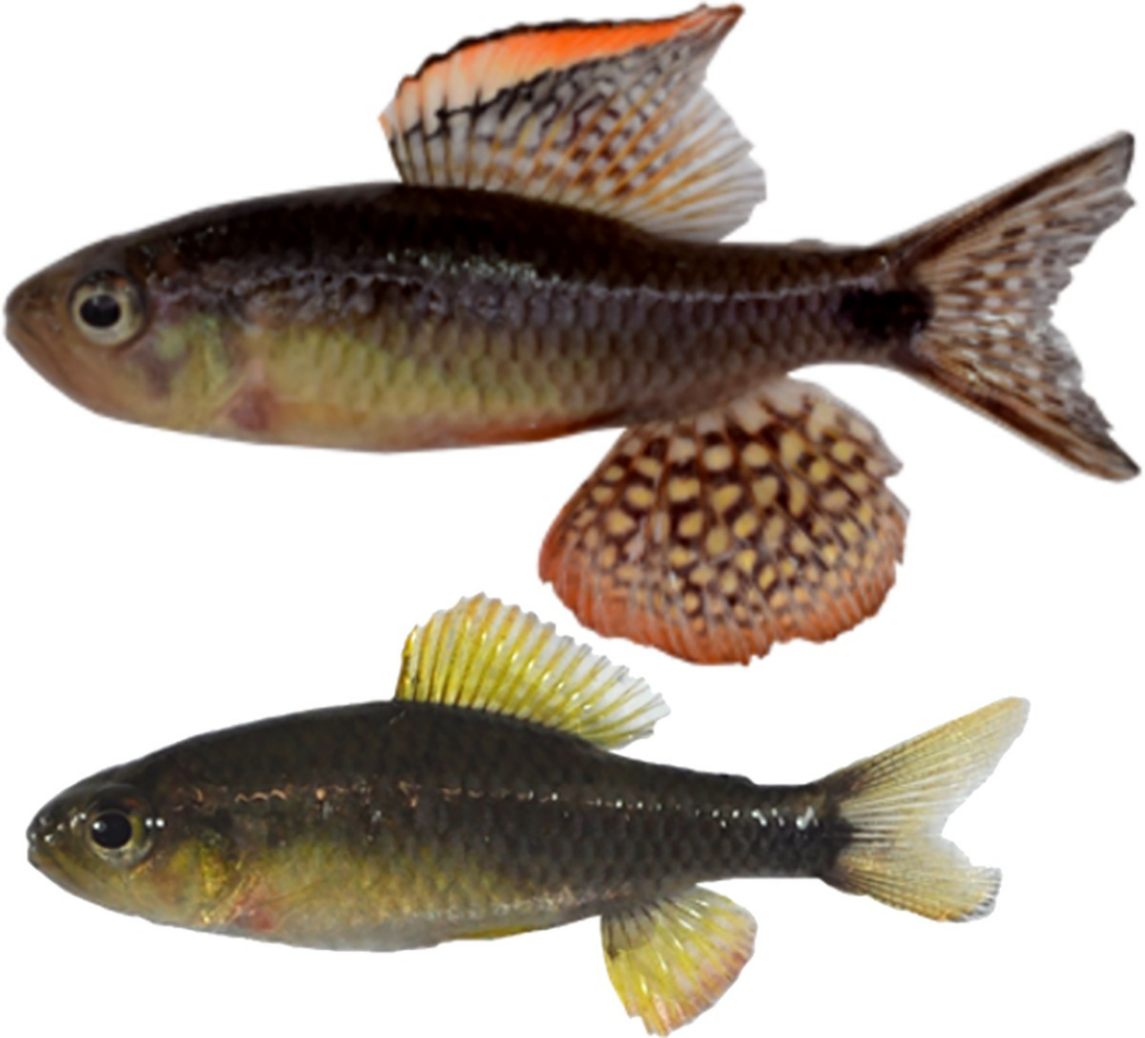
*Crenuchus spilurus* individuals. Male above and female below. Note differences in size, shape and color of the anal and dorsal fins.

The sailfin tetra *Crenuchus spilurus* (Characiformes: Crenuchidae) is a sexually dimorphic and dichromatic Amazonian fish species (Fig 1). This species occurs in forest streams of semi-lentic and stable environmental conditions [56]. Males possess hypertrophied anal and dorsal fins that are conspicuously ornamented in red and yellow. Males spread the fins during a long and complex courtship behavior, which can last for several consecutive days [56]. Ornaments are also exhibited in male-male contests. Males exert exclusive paternal care for circa eight days, until the larvae detach from the nest substrate, whereas females seem not to play any important role in parental care [56]. During this period, males rarely leave the nest and refrain from feeding [56], which also decreases opportunities for courtship activity. Because males exert exclusive paternal care and females invest more energy per gamete, mutual mate choice could be expected in the sailfin tetra. Here we investigated the relationship of male and female body size with their respective conspicuousness and fecundity. Additionally, we asked whether males and females prefer larger partners or a preference for similar-sized partners was prevalent. We addressed these questions by investigating male and female choice and spawning success through controlled experiments in captive conditions.

## Material and methods

### Ethics statement

This study was conducted according to national laws about animal care and was approved by Comissão de Ética no Uso de Animais (CEUA) of the Instituto Nacional de Pesquisas da Amazônia (INPA), under protocol number #029/2016. Field work was authorized by Divisão de Suporte às Estações e Reservas (DISER) of the INPA. Fish sampling was carried out under IBAMA permanent license SISBIO 10199–1 to JZ and following local regulations.

### Biological system

Larger sailfin tetra males are usually dominant and remain inside nesting sites, whereas smaller males actively search for females (personal field observations by E.A.B. and T.H.S.P.). During courtship, males attempt to conduct them to nesting sites, which are composed of hard substrate such as dead leaves and rocks [56]. Females often swim inside such nesting sites, but it remains unclear whether this represent a form of nesting site inspection that might indicate assessment of male quality. Sailfin tetra is a non-seasonal breeder, as females show multiple egg batches and can spawn several times during a year [56]. Couples can spawn multiple times throughout a year in captivity [56]. During field observations, only one male was found tending for eggs at different developmental stages [56], which suggests that, although not frequent, males can spawn with more than one female in a single reproductive event (i.e. polygyny).

### Sampling and fish husbandry

Individuals of *Crenuchus spilurus* were collected from an urban forest fragment in central Amazon (3°6’22.94”S 59°58’42.48”W). These individuals belong to the N1 population of the Rio Negro lineage [58]. To increase sexual receptivity, fish were separated by sex for two months before experimental procedures started. All fish were housed in 92 liters tanks, each containing individual filters and air pumps. Water changes were performed weekly to maintain water quality. A 12–12 light/dark regime and a constant temperature of 24 ^o^C were maintained to simulate natural habitat characteristics. All individuals were fed high quality commercial fish food once a day. Individuals in mate choice experiment were used only once. We included only adult individuals in the study, as all individuals were larger than 2.57 cm, the estimated size-at-maturity for the species [56].

### Ornaments, fecundity and egg size

We photographed 26 adult males (with recognizable sexual ornaments) to obtain measurements of ornaments. We first anesthetized the fish in eugenol solution (0.5 ml per liter of water). Upon noticing a reduction in swimming activity, we placed the fish on a white paper next to a scale, spread the dorsal and anal fins as much as possible while avoiding damage to the membrane using a soft brush and prevented fins from folding using entomological needles [59]. Pictures were taken using a photo camera (Nikon D90 with 50mm lenses) attached to a standing pole that maintained a standard distance to the fish. We extracted three measurements for each individual: standard length (SL: from snout tip to caudal fin peduncle, i.e. length of fish not including caudal fin) and the areas of the anal and dorsal fins. We conducted the same procedure and measured SL, and the dorsal and anal fin areas for 23 adult females. Because dorsal and anal fins in females are mostly transparent, we assumed that the role these fin areas of females is unrelated to sexual selection (i.e. exclusively controlled by natural selection). The differences in fins areas of males in relation to females are considered the investment in conspicuousness of ornaments, as opposed to increased investment in swimming stability. All measurements were taken using the software ImageJ v1.3.

Females were euthanized in a lethal concentration of eugenol solution (8 ml/L) and preserved in formalin for fecundity measurements. We included only females larger than 30 mm SL into analysis, since females reach sexual maturity at 27.5 mm SL [56]. Fecundity and mean oocyte size were estimated for each female considering only the most developed batch (i.e. oocytes in later stages of development) [56]. Only ripe (vitelogenic) oocytes were counted and measured on stereomicroscope (N = 14 females). Fecundity was measured using the gravimetric method [60,61] and mean oocyte size was calculated as the average size of all oocytes from the most developed batch.

### Dichotomous choice

An experimental tank (70 x 35 x 35 cm, ~73 liter of water) was segmented into three compartments in which chemical communication was constrained using two acrylic plates: two lateral compartments measuring 20 cm and a central compartment of 30 cm of length. The central compartment was divided into three parts by two imaginary lines 10 cm away from the acrylic plates and toward the central region of the tank. The two areas near the lateral compartments were considered association zones to the offered stimuli (males or females of distinct sizes), a set-up commonly used to measure preference in fish [43,62,63]. To minimize interference from visual stimuli of the exterior, experimental tanks were isolated from other parts of the experimental room by two layers of black cloth.

**Fig 2:**
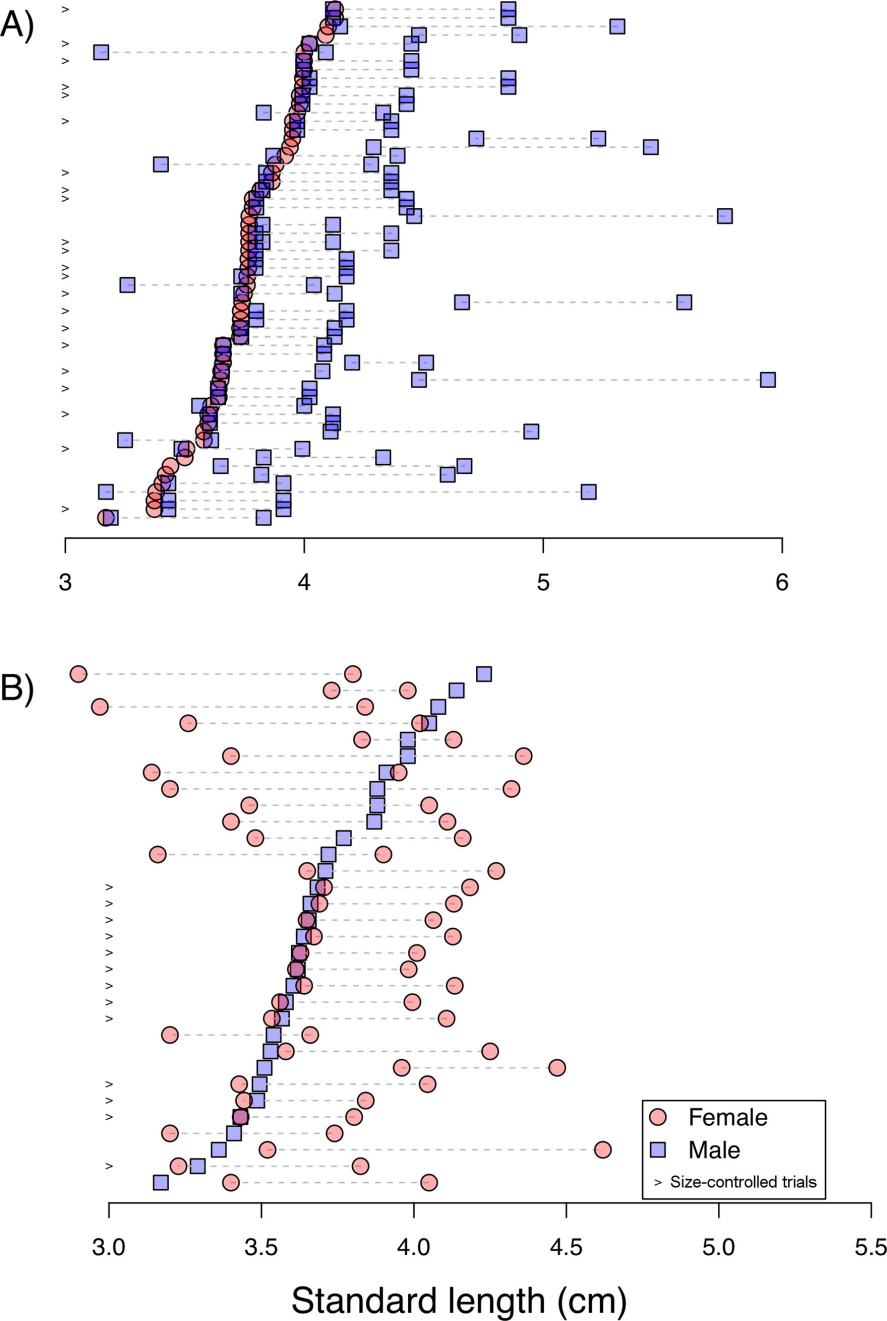
Graphical representation of sizes of individuals used in dichotomous choice trials. Blue squares represent males, red circles represent females used in A) female choice trials, and B) male choice trials. The > symbol represents trials using size-controlled individuals, other trials involved random selection of fish sizes.

To measure female choice for larger mates, we initially placed opaque black plates near the acrylic plates, two random males of visually different sizes were inserted into the lateral compartments (size of trios used in the experiment are represented in Fig 2), and a female (selected randomly regarding body size) was placed in the central compartment. The only requirement to the mate choice experiment was that offered males and females on both lateral compartments were of visually different sizes. Thus, in female mate choice trials, females could be similar in size to the larger or to the smaller male; the same applies for male mate choice trials. To capture fish from the stock tanks, we initially moved a dip-net in a circular movement to displace fish from the vicinities of the filter, the region of the tank where they seek shelter when disturbed. We then captured the fish that was closest to one of the tank walls. This procedure assured that capture of fish was random in respect to body size. After being captured, fish were placed on a glass surface and we used a digital caliper to measure its standard length (SL). After an acclimation period of one day, we gently removed the black plates and recorded movement of the fish for one hour using an overhead camera placed 1.3 m above the tank. The same procedure was repeated to measure preference of males for females of different sizes.

Because body size among focal and choice individuals was initially randomized, difference in body size among these individuals highly varied in trials. Therefore, to test female and male preference for similar-sized mate partner we conducted a second set of trials, in which one choice individual was similar-sized to the focal individual. We conducted the same procedure described above, but applied a greater control on the size of presented partners. Each focal fish had the choice between a similar-sized opposite-sex individual that was distinct in 6 mm or less, and a dissimilar individual that was at least 35 mm larger than the focal individual.

The position of the fish within each frame of the video was extracted using the open-source software Swistrack [64]. For each video, we first extracted several frames using FFmpeg v3.3, which were read in GIMP v2.8 for the creation of background images. Background images consist of an image of the experimental setup captured by the camera, excluding only the objects that will be tracked; in our case, the fish. Once the positions of the fish were extracted, we read the values in R [65] and used a custom script to automatically measure time, in seconds, spent by fish in each association zone (= association times), which represented preference for opposite-sex fish of different sizes.

### Spawning success

We conducted an independent test to evaluate spawning success using isolated couples. Isolated couples were used because aggressive interaction between males can constrain female choice [66], as dominant males prevent subordinated males from courting or accessing the nesting site. We reasoned that an agreement between the independent experiments (preference in dichotomous choice trials and spawning success) would be indicative that dichotomous choice trials are a reliable measure of reproductive success in the sailfin tetra, as inference of choice based solely on dichotomous choice trials have limitations [67]. Mate preference may be stronger when evaluated in choice experiments [66,68]. Our mate choice experiment was performed using a simple design in which both stimuli could see each other. Visual interactions can potentially affect the establishment of hierarchical structure, which may affect stimuli responses and consequently the chooser individual association. More importantly, previous studies have questioned the validity of dichotomous choice experiment to evaluate mate choice [69,70]. Therefore, in order to assess whether the results from the dichotomous choice experiment actually translated into actual mate intentions we conducted an experiment to measure spawning success of couples using fish that were not previously exposed to any experimental condition.

We collected dead leaves from a natural sailfin tetra forest stream habitat and added them to the water used in our experiment to create a natural water condition. The plant material was left to leach for 10 days, and 1 kg (wet weight) of plant material was used per 100 l of water, in order to increase spawning success rate [71]. We set couples into individual tanks (40 x 30 x 30 cm), each containing two spawning sites (PVC pipes 10 cm long and 2.5 cm in diameter) and an artificial plant. Couples were fed once a day in the morning and the interior of the PVC pipes were inspected for presence of eggs using a flashlight. This procedure was repeated for a maximum of 20 days. If no egg was detected, the trial was registered as having no spawning success. When eggs were detected, the trial was registered as a successful spawning event of the couple. Individuals were used only once in our spawning success experiment. Tank water was completely renewed each day via a flow-through system exchanging tank water for new water at a rate of 1.5 l/h. All fish were measured using a digital caliper (in SL) before being transferred into the tanks.

### Statistical procedures

The areas of the dorsal and anal fins of both males and females were regressed against body size (in SL) using nonlinear power regressions (*Y = a·SL*^*x*^). We fitted these models to infer the exponent (x) and its significance value. This approach was favored over traditional analysis of allometry as this approach models only the error associated with area. This is important since in our behavioral trials, we only measured body length, so that this model gives the expected size of fins for given value of standard length. We reasoned that the relationships of fin areas and body size of females could be considered as stemming solely from natural selection: fins are maintained by natural selection on swimming stability. On the other hand, the relationship between fin area and body size of males is driven by combined contribution of sexual and natural selection: fin areas result from natural selection on swimming stability and sexual selection as ornaments. Therefore, the difference in values of the curves when evaluate body size as a function of area of ornaments of males and females are considered the increase in conspicuousness of males resulting solely from sexual selection. We also used nonlinear power regression to investigate variation in female fecundity and oocyte diameter with female body sizes. This procedure allowed calculation of the exponent, which represented the rate of potential fitness increase with size (assuming a relationship between fitness and fecundity and oocyte sizes). Ordinary linear regression would mask out the potential escalating importance of body size in fitness as measured by direct fecundity parameters.

To test preference for larger mates, we used the first dichotomous choice experiment dataset (with randomized body size) and for similar-sized mates we used the second one (controlling body size). In each dataset, we conducted a paired t-test on raw association times to investigate preference for the larger of the two and for similar-sized offered opposite-sex individuals. We excluded trials in which the focal individual failed to spend more than 20% of the association time with one of the offered individual (seven trials), and trials in which one of the three individuals remained stationary for more than 30 minutes (four trials). In all trials the focal individual visited both association zones.

For the spawning success experiment, we conducted a logistic regression with spawning success as the dependent variable and absolute size difference of couples as the independent variable. All data can be found in S1–S7 Files.

## Results

### Ornaments and fecundity

**Fig 3:**
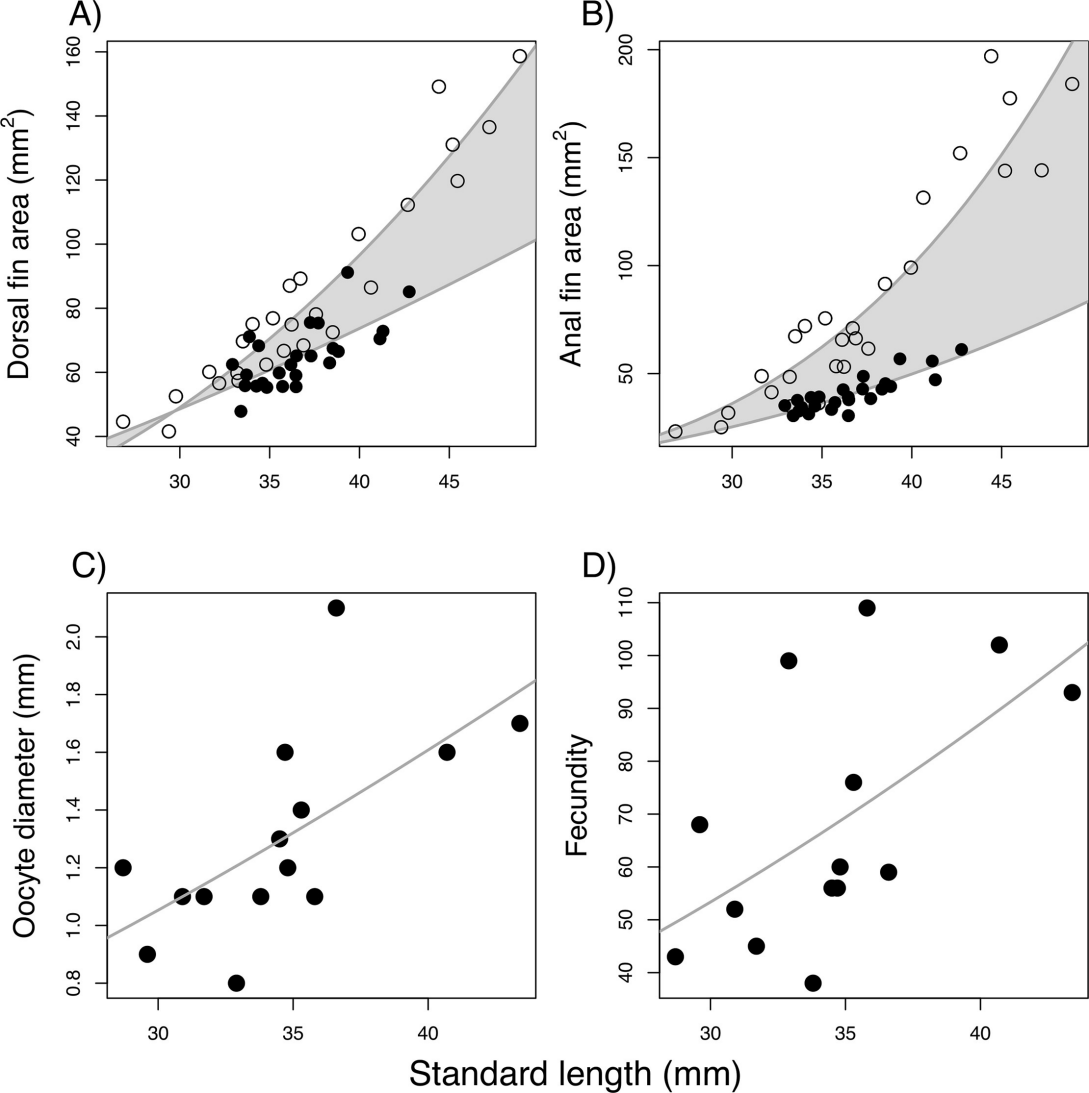
Relationship between measures of individual quality and body size in *Crenuchus spilurus*. The function of (A) dorsal and (B) anal fins as ornaments increases as males grow. Females are represented by filled circles and are taken as a baseline measure of the fin function as swimming stability (driven solely by natural selection). Difference between curves (grey shaded areas) represents the function of male’s fins as ornaments. The cone format of this shaded area (with base toward highest values of SL) indicates an increase in the function as ornaments of male’s fins as individuals grow. Reproductive investment of females increases with size, as observed by the positive relationship of oocyte size (C) and fecundity (D).

As expected, the area of dorsal and anal fins increased with size of males and females (Table 1). As individuals increased with body size, conspicuousness of the dorsal and anal fins of males increases, as does their likely function as ornaments. This can be observed by the cone-shaped shaded area of Fig 3A and 3B. Similarly, as females increased with body size, the number and size of the oocytes increase (Fig 3C and 3D, Table 1).

**Table 1:** Inferred parameters and significance values of exponent (x) power regression (*Y = a·SL*^*x*^) conducted to measure the relationship of several biotic characteristics against standard length (SL) of *Crenuchus spilurus*. Regression lines are drawn in Fig 1.

	*a*	*x*	*P*
Dorsal fin			
Males	0.01	2.35	<0.001
Females	0.35	1.45	<0.001
Anal fin			
Males	2.10^−4^	3.35	<0.001
Females	0.008	2.37	<0.001
Fecundity	0.16	1.7	<0.05
Oocyte size	0.007	1.47	<0.005

### Mate choice

**Fig 4:**
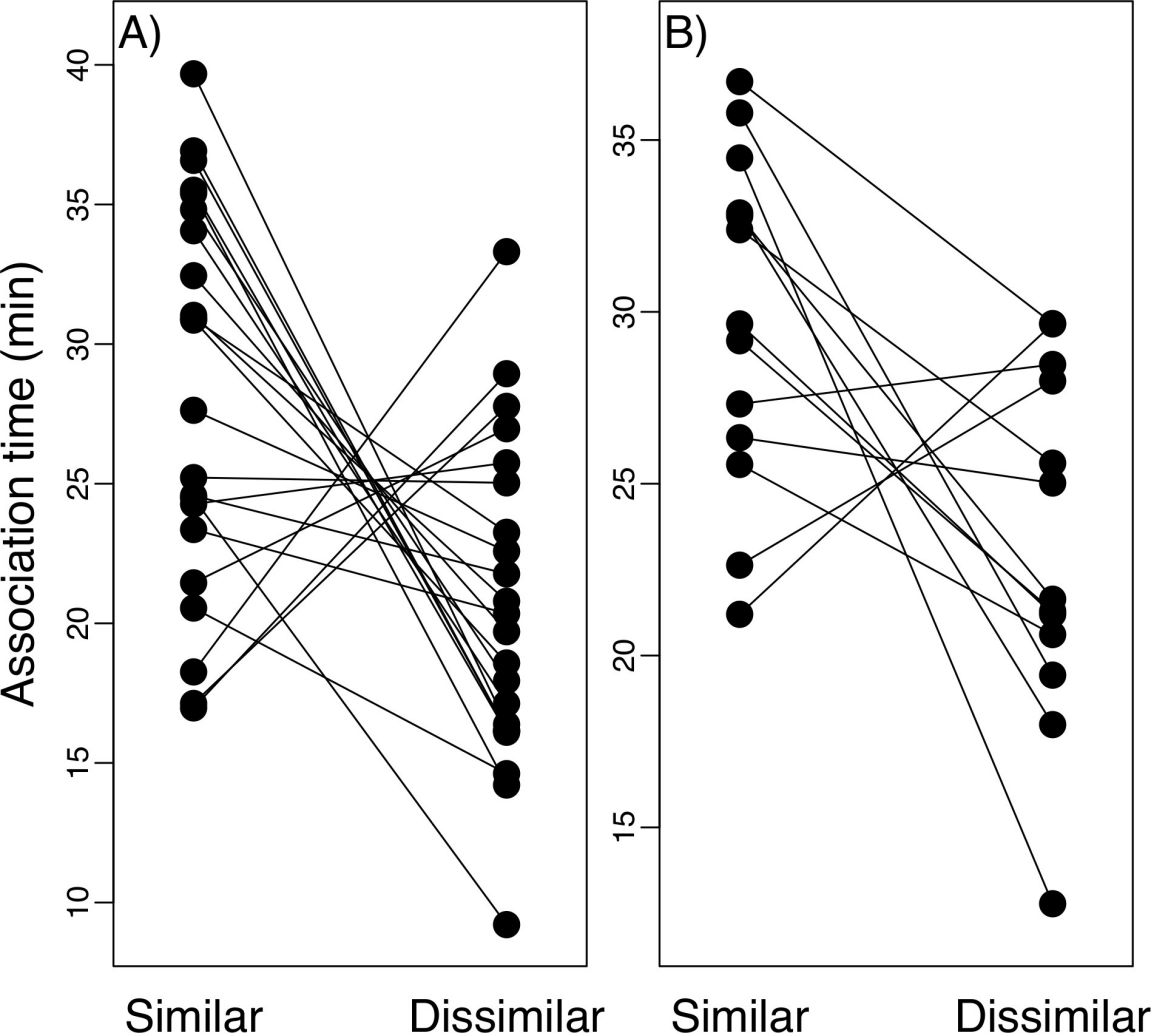
Association time of according with size-similar and size-dissimilar individuals. Trials conducted under (A) female choice experiment, and (B) male choice experiment. Lines connect observations from the same individual.

We obtained results for 19 males and 38 females tested using the dichotomous choice trials using random sampling of individuals from stock tanks. Males did not associate longer with the larger of the two offered females (t-test, t = -1.89, P = 0.07). Females, showed a statistically significant longer association time with the smaller of the two offered males (t-test, t = -3.32, P<0.005). Mean association time of females with the smaller of the two offered males was 29.71 minutes as opposed to 21.32 minutes with the larger male. We obtained results for 22 females and 13 males in dichotomous choice trials designed to test preference for similar-sized mates. Both females (Fig 4A) and males (Fig 4B) associated longer with the most similar individual presented to them (paired t tests: t = -3.13, P = 0.005; t = -2.77, P = 0.01).

### Spawning success

**Fig 5:**
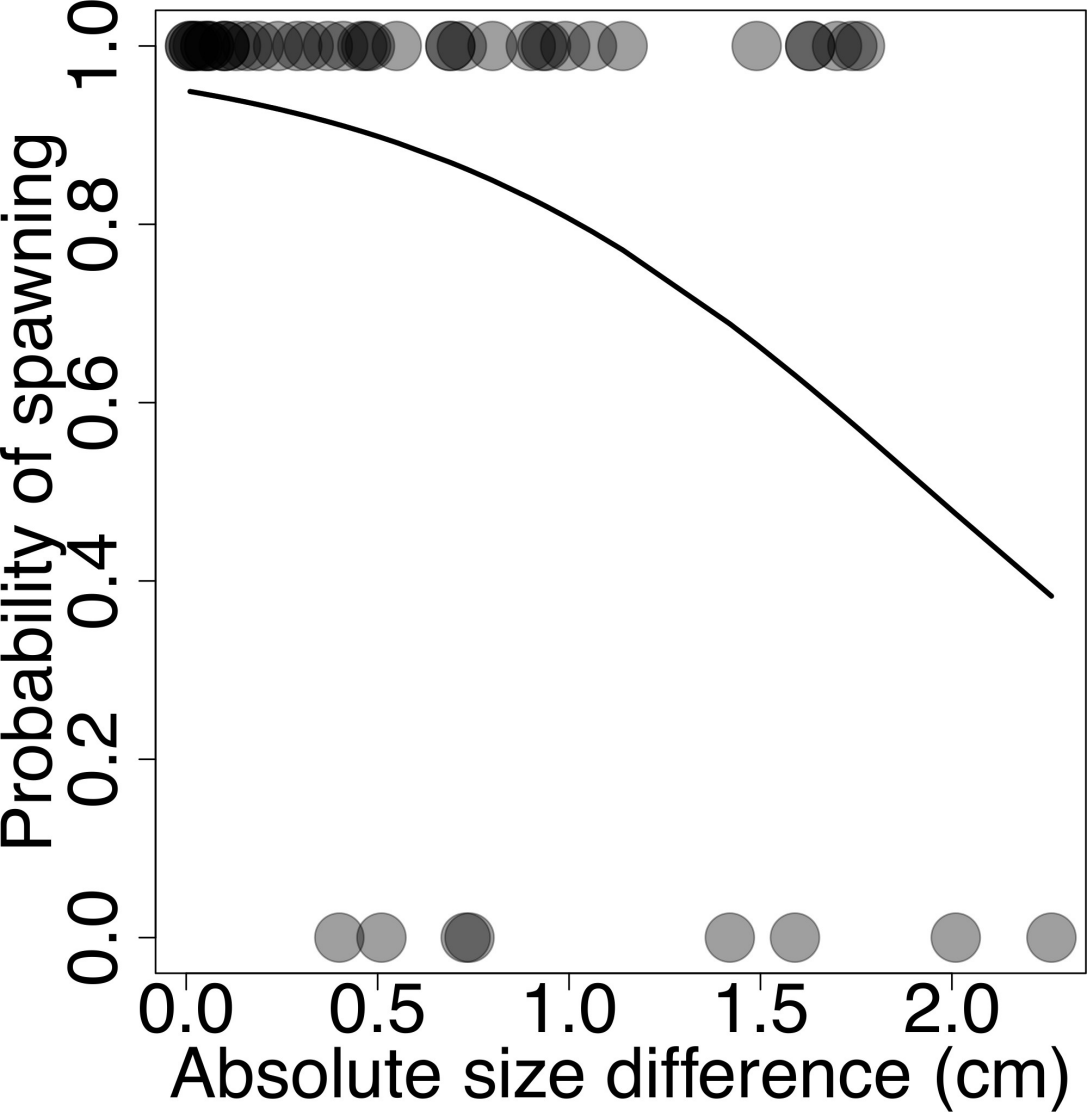
Spawning success in relation to size asymmetry of sailfin tetra couples. Males and females more similar in body length (lower size asymmetry), are more likely to spawn than couples with larger size asymmetries. Shades of gray represent number of trials with similar size asymmetry (darker points represent more trials).

We assessed spawning success for 48 couples, 40 of which successfully spawned in our tanks. We found a significant negative relation between spawning probability and absolute size difference (Z = -2.35, P = 0.018, Fig 5), suggesting that similar-sized couples were more likely to spawn.

## Discussion

Despite having traits that are indicative of higher quality and attractiveness, larger opposite-sex individuals were not preferred by neither males nor females in our study. Instead, females and males associated longer with individuals of the opposite sex that were more similar in size to their own. This choice seemed to reflect actual mating decisions, with similar-sized couples being more likely to spawn than couples composed of male and female of greater size asymmetries. Our results reject absolute preference for larger partners, which is expected under preference for high mate quality regarding aesthetics, fecundity and resource dominance. We suggest that mate choice in *Crenuchus spilurus* is better explained by a self-referent preference [72], which poses that constraints are central in shaping evolution of mate choice.

Female choice for larger males is common in nature [1,25,28,31,73]. Larger males commonly have higher competitive abilities, being frequently dominant and more capable of holding resources [21,22,24]. Although exceptions to this general pattern may occur [74], such instances seem to be restricted to species with polygynous mating systems in which rate of encounter with potential mates is high, which seems not to be the case of the sailfin tetra. Mutual mate choice is expected when males exhibit extensive parental care, as they become incapable of investing in new mates for long periods [19,35,36]. Sailfin tetra males undertake exclusive paternal care, a period of circa eight days in which males rarely leave their nest [56]. Also, males invest several days in courtship behavior before the spawning event [56], which may increase their choosiness. We have little evidence of polygyny for this species, as only one clutch found in the field contained eggs at two different developmental stages, however it is unclear if they were both laid by the same female [56]. Mutual mate choice in the sailfin tetra may result from a somewhat balanced parental investment between sexes of the sailfin tetra. In *C*. *spilurus*, males expend high energy in parental care, and females follow the most typical pattern in nature, with high parental investment in the form of much larger gametes [20].

Under the pure aesthetic preference originally put forth by Darwin [29], females are expected to choose males that bear more conspicuous ornaments [30,62,75,76], which for many species is seen in larger males [33]. The function of the dorsal and anal fins as ornaments in males of the sailfin tetra increases with size, with a corresponding increase in conspicuousness (Fig 1A and 1B). Moreover, larger males of the sailfin tetra are dominant in social groups, indicative of higher competitive ability. Therefore, we hypothesize that females would prefer larger males. However, contrary to expectations considering such potential benefits, females did not prefer the larger of the two offered males in our dichotomous choice trials and were more likely to mate with similar-sized males in our no-choice experiment.

We showed that in the sailfin tetra, as expected for a fish species, size of oocytes and fecundity increases with size of female *C*. *spilurus* (Fig 1C and 1D, Table 1). In fish and other indeterminate growth organisms, males are expected to prefer larger females, given the adaptive advantage of mating with females of higher fecundity and larger eggs, which is related to number and quality of offspring [1,38]. However, despite such potential fitness benefits, males of the sailfin tetra did not associate longer with larger females.

Mutual mate choice in *C*. *spilurus* seems to be best explained by the prudent mate choice hypothesis [53], which posits that mate preference is shaped by a trade-off between the competitive ability of the choosing individual and quality of potential mates. Under this hypothesis, size-assortative mating emerges from optimal choice of mates that is exerted in a compromise between pairing with a female of the highest possible quality given the limitations of competitive ability of the choosing male [51,77,78]. As such, males actively choose to initiate courtship activities towards similar-sized females, a decision that reduces the chances of mate takeover by larger males, which will preferably pair with larger females. In the sailfin tetra, males initiate courtship activity by swimming toward females and displaying ornaments. Courtship activities of the sailfin tetra are very long, and courtship movements performed by the same pair can extend up to seven consecutive days [56,79]. This long pair-bonding courtship process should increase the chances of female takeover and limit the ability of low-quality males to keep a high-quality female until spawning. Indeed, during direct field observations conducted for a previous study, takeover of females by larger males during courtship was observed in all (eight) occasions in which a female was being conducted to a nesting site by a male (S1 Video). Therefore, the decision of male *C*. *spilurus* to pair and court females of similar size should limit the chances of takeover and reduce time and energy costs of attempting to maintain higher qualities mates. Importantly, the observed takeover cases never involved fighting, so that courting high quality females does not seem to commonly incur in physical injuries to males.

In a similar fashion to the process suggested for males *C*. *spilurus*, female choice for similar-sized males in *C*. *spilurus* could also result from a compromise between maintaining male and female competitive abilities [78,80]. Indeed, females favoring males of similar quality to their own has been previously reported and suggested to be mediated by intrasexual competition [9]. However, we have no evidence favoring this process in the sailfin tetra, as no case of male takeover by a female was observed in the field. We speculate that female mate choice might have evolved in response to sexual conflict due to filial cannibalism by males [81]. Egg predation by males in species that exert exclusive paternal care is common and widespread among fishes [82] and the sailfin tetra is no exception. Filial cannibalism might assist paternal care by providing energy to the male or it may represent a trade-off between investment in current and future reproduction [81]. This trade-off seems particularly important for the sailfin tetra, as males undertake a long (circa eight days long) period of exclusive paternal care in which they do not feed [56]. As such, large *C*. *spilurus* males might benefit from the energy acquired by the complete consumption of the egg clutch laid by a small female and progress to pursuit a larger (more fecund and carrying larger eggs) female [83,84]. The amount of eggs laid by a small female partner might not be enough to compensate the energy expenditures required for the long period of parental care males *C*. *spilurus* undertake, increasing the likelihood that a large male will completely consume an egg clutch laid by a small female. Females can thus experience a trade-off between the benefits from having a large partner with the likelihood of filial cannibalism. By choosing males of similar sizes of their own, females would be minimizing the chance of egg predation by the male after spawning while keeping males of high quality in terms of the benefits provided by large body size. We expect female mate choice to be more complex than what is portrayed for males by the prudent choice hypothesis, thus demanding a specific study to assess the relative importance of each potential mechanism behind the pattern of preference for similar-sized partners here described.

In our mate choice trials, females and males were kept in different tanks for two months, and have thus experienced a highly-biased sex ratio prior to experimentation. This is expected to affect our results if mate choice is mediated by competition with same-sex individuals [12]. For instance, males could choose larger females in the absence of prior contact with larger males, since the chance of female takeover would be reduced. This raises an interesting possibility of future mate choice studies in *C*. *spilurus*. Since we raise the possibility that size-assortative male choice results from competition with other males, and we also suggest that female choice for similar-sized males results from intersexual conflict, it can be predicted that manipulating the experienced sex-ratio before trials would affect male mate choice but not female choice. Predation risk and physiological state of individuals can affect mate preference and were not considered in our study. Under high predation risk, individuals may adjust their preferences reducing or even reverting preference for conspicuous mates [85–88]. By the same token, mate preference may vary according to female reproductive cycle [67]. Future studies may shed light on context-dependent variation in mate choice and relationship between reproductive cycle, predation risk and mate choice.

It has been reported that females may induce agonistic encounters between males in order to assess the quality of potential mates [66]. By actively swimming away from the male during the long courtship process, females *C*. *spilurus* might induce contests between males. However, such swimming movements might also indicate an attempt of the female to direct the male near a nesting site. Investigating such interesting aspects are beyond the scope of the present study.

The use of dichotomous choice trials and association times might be problematic as they can measure choice of social partners, failing to assess actual mating decisions [69,70]. Here we acknowledged this skepticism and avoided drawing conclusion based solely on this dubious measure of choice. This motivated the investigation of actual mating in the sailfin tetra using isolated couples. Based on agreement between results of association times and mate choice experiments (indicating size-assortative pairing and mating), we suggest that mate choice as measured by association times reflects both active social pairing decision and actual mating in the sailfin tetra, a pattern also observed in other studies [63,73].

## Conclusion

Although larger individuals represent a higher fitness potential, neither males nor females display clear choice for larger individuals. Instead, we found evidence that individuals of both genders actively choose similar-size individuals as mating partners. Based on field observations, mate choice in the sailfin tetra seems to be constrained by intrasexual selection, via mate takeover among males, and possibly by filial cannibalism. Importantly, our study evaluates mating preference in two independent experiments and the results found agree with one another, suggesting that mating choice evaluated through dichotomous choice experiment reflect actual mating intention in the sailfin tetra.

## Supporting information

S1 Video Takeover of a female by a larger male during courtship in field conditions. A small male is courting a female while a larger male approaches and drive out the smaller one. After acquiring female attention, larger male swims toward a PVC pipe (provided by us) which simulates natural spawning sites.
(MP4)

S1 File Relationship between fins and standard length between males and females. Sex = Individuals sex; CP = Standard length; AA = Anal fin area; AD = Dorsal fin area.
(CSV)

S2 File Female fecundity and egg size. ID = female Identity; CP = female standard length; Fecundity = female fecundity; Oocyte.size = oocyte size.
(CSV)

S3 File Female dichotomous choice with body size randomly selected. female_size = female standard length; size_preferred_male = standard length of the preferred male; size_rejected_male = standard length of the rejected male; time_near_larger_male = time near to the larger of the offered males; time_near_smaller_male = time near to the smaller of the offered males.
(CSV)

S4 File Male dichotomous choice with body size randomly selected. male_size = male standard length; size_preffered_female = standard length of the preferred female; size_rejected_female = standard length of the rejected female; time_near_larger_female = time near to the larger of the offered females; time_near_smaller_female = time near to the smaller of the offered females.
(CSV)

S5 File Female dichotomous choice with controlled body size. Time = time in association zone with a specific stimulus individual; Female.size = female standard length; Male.size = male standard length; id = trial identity.
(CSV)

S6 File Male dichotomous choice with controlled body size. Time = time in association zone with a specific stimulus individual; Male.size = male standard length; Female.size = female standard length; id = trial identity.
(CSV)

S7 File Spawning success. Couple = trial; male.size = male standard length; female.size = female standard length; spawning = occurrence of reproductive events; size.dif = difference between male and female size; abs.size.dif = absolute difference between male and female size.
(CSV)
